# The Living-Out System

**Published:** 1920-12-25

**Authors:** 


*To the Editor of* The Hospital.

### "The Living-out System."

*To the Editor of* The Hospital.

Sir,—Your correspondent "Cornubia" asks, what salaries would be considered adequate by the advocates of the living-out system?

As partial board would be provided, I would suggest staff nurses should commence at £210 per annum, rising by a yearly increment of £10 to £300; sisters to commence at £300, rising by a yearly increment of £15 to £400.

The living-out system would be an inducement to nurses to work in the vicinity of their homes, and it would also be a further inducement to trained women to run hostels on the principles laid down by "Cornubia" for professional women, not necessarily nurses alone. In many districts one hostel could serve the needs of more than one non-resident nursing staff. Unfortunately, to-day there are few professional women who can view the future from an economic standpoint without anxiety, and if progress is to be dependent on an assured financial outlook, we are not likely to advance for many years to come.—Yours faithfully, The Writer of the Article.

